# Epidemiology of Cryptosporidiosis in France from 2017 to 2019

**DOI:** 10.3390/microorganisms8091358

**Published:** 2020-09-04

**Authors:** Damien Costa, Romy Razakandrainibe, Stéphane Valot, Margot Vannier, Marc Sautour, Louise Basmaciyan, Gilles Gargala, Venceslas Viller, Denis Lemeteil, Jean-Jacques Ballet, French National Network on Surveillance of Human Cryptosporidiosis, Frédéric Dalle, Loïc Favennec

**Affiliations:** 1 Department of Parasitology/Mycology, University Hospital of Rouen, 76000 Rouen, France; gilles.gargala@chu-rouen.fr (G.G.); denis.lemeteil@chu-rouen.fr (D.L.); loic.favennec@chu-rouen.fr (L.F.); 2 EA ESCAPE 7510, University of Medicine Pharmacy Rouen, 76000 Rouen, France; romy.razakandrainibe@univ-rouen.fr (R.R.); balletjeanj@laposte.net (J.-J.B.); 3 CNR LE Cryptosporidiosis, Santé Publique France, 76000 Rouen, France; Venceslas.villier@univ-rouen.fr; 4 CNR LE Cryptosporidiosis Collaborating Laboratory, Santé Publique France, 21000 Dijon, France; stephane.valot@chu-dijon.fr (S.V.); louise.basmaciyan@chu-dijon.fr (L.B.); frederic.dalle@chu-dijon.fr (F.D.); 5 Department of Parasitology/Mycology, University Hospital of Dijon, 21000 Dijon, France; Marc.Sautour@u-bourgogne.fr; 6 Department of Biostatistics, Rouen University Hospital, 76000 Rouen, France; Margot.Vannier@chu-rouen.fr

**Keywords:** cryptosporidiosis, epidemiology, France

## Abstract

Cryptosporidiosis is currently recognized worldwide as a leading cause of moderate to severe diarrhea. In Europe, large water- and foodborne outbreaks have been reported, highlighting the widespread distribution of the parasite and its important health impact. Surveillance networks have been progressively set up and the aim of this study was to present recent epidemiological data obtained in France from 2017 to 2019 by the National Reference Center—Expert Laboratory of cryptosporidiosis (Centre National de Référence–Laboratoire Expert cryptosporidioses CNR-LE). Data were obtained from online reports of volunteer network participants and stools were sent to the CNR-LE for species identification and *GP60* genotyping. During this period, data from 750 online reports were available. Cryptosporidiosis occurred predominantly in young children (<5 years old) and in young adults, especially during late summer. Most patients were immunocompetent (60%), and deaths were reported only in immunocompromised patients. *Cryptosporidium parvum* was largely predominant (72% of cases) over *C. hominis* (24%) and some other uncommon species. *C. parvum GP60* subtypes IIa and IId were the most represented, which suggests frequent zoonotic transmission. For *C. hominis,* subtypes IbA10G2 and IaA22R2 were predominant.

## 1. Introduction

Among Apicomplexa, *Cryptosporidium* spp., the only genus of the Cryptogregaria subclass, is recognized as a major foodborne parasite [1,2]. The Global Enteric Multicenter Study (GEMS) revealed that *Cryptosporidium* was the second leading cause (5–15%) of moderate to severe diarrhea in infants in countries of sub-Saharan Africa and South Asia while 60,400 deaths due to *Cryptosporidium* spp. (representing 12.1% of deaths among children younger than five years with diarrheal disease) were reported worldwide in 2015 [3,4]. At the same time, *Cryptosporidium* was responsible for more than 8 million cases of foodborne illness in 2010 and was ranked fifth out of 24 potentially foodborne parasites in terms of importance [2,5]. 

Directly infective oocysts are shed in stools of contaminated hosts and can infect new hosts via fecal-oral transmission. Nowadays, at least 39 *Cryptosporidium* species have been described [6]. All are not encountered in humans and depending on species, a diversity of host range is observed. Regarding humans, two species are responsible for the large majority of infections: *C. parvum* and *C. hominis*. *C. parvum* is known to be able to infect a large diversity of hosts including humans, ruminants, rodents whereas *C. hominis,* which was considered for a long time specific to humans, is currently observed in other hosts such as ruminants [6,7,8,9,10]. More rarely, and thanks to the development of molecular epidemiology tools, other species have been identified in humans (especially in those with any kind of immunodepression): *C. xiaoi, C. felis, C. meleagridis, C. canis, C. erinacei, C. cuniculus, C. viatorum,* and *C. occultus* [6,11,12]. 

The low infective dose (calculated at 132 oocysts in healthy volunteers for *C. parvum* and 10 to 83 oocysts for *C. hominis*) [10,13,14,15], ubiquitous distribution, and resistance to disinfectants [16,17] have led to the frequent implication of *Cryptosporidium* in food/waterborne outbreaks despite under-reporting due to the lack of adequate detection and surveillance systems [5,18,19,20]. In France, there is no incentive to perform systematic cryptosporidiosis screening in diarrheic patients and no obligation to report cryptosporidiosis cases. It was only in 2006 that a tentative *Cryptosporidium* National Network was supported by the French authorities to monitor the national epidemiology of cryptosporidiosis and finally, in 2017, the Network was recognized as the National Reference Center-Expert Laboratory (CNR-LE). The aim of this study was to describe the characteristics of cryptosporidiosis cases observed in France and reported to the CNR-LE from 2017 until 2019. 

## 2. Materials and Methods 

According to guidelines available online on the CNR-LE website, cryptosporidiosis reports forms are available to a network of French clinical laboratories: https://cnrcryptosporidioses.chu-rouen.fr/espace-professionnel/declaration-des-cas/. Notified cases are either (i) already confirmed using a diagnosis method chosen by the reporting laboratory which sent (if possible) the stool sample or DNA to the CNR-LE; (ii) or not confirmed. In the latter case, stool samples are sent to the CNR-LE for expertise, and online case reports are made secondarily if cryptosporidiosis is confirmed by the CNR-LE. 

The number of laboratories participating in the network increased from 43 tertiary care public hospitals and three private laboratories in 2017 to 49 tertiary care public hospitals and 15 private laboratories in 2019. Public hospitals included all French university hospitals and some additional non-university hospitals, and covered the entire territory of France, including all mainland regions as well as overseas territories. In contrast, for private laboratories, four French regions were not represented: Bourgogne-Franche-Comté, Centre-Val de Loire, Corse, and Occitanie.

A total of 750 online reports were available from 2017 to 2019. Notified items were: age, sex, immune status, date of diagnosis, symptomatology, therapy, clinical evolution, location, and exposure to risk factors.

DNA was extracted from received samples using the QIAamp PowerFecal DNA kit (Qiagen, courtaboeuf, Hilden, France) according to manufacturer’s instructions. Firstly, a real-time PCR was performed to evaluate the presence of *Cryptosporidium* species according to the protocol described by Hadfield et al. and Brunet et al. [21,22]. GP60 subtypes were identified according to the protocol described by Sulaiman et al. [23]. A nested PCR was performed using primers: AL3531 (5′-ATAGTCTCCGCTGTATTC-3′)/AL3533 (5′-GAGATATATCTTGGTGCG-3′) and secondly AL3532 (5′-TCCGCTGTATTCTCAGCC-3′)/LX0029 (5′-CGAACCACATTACAAATGAAGT-3′). Thermocycling conditions were: 94 °C for 3 min, followed by 40 cycles of 94 °C for 45 s, 54 °C for 45 s and 72 °C for 60 s and a final step at 72 °C for 7 min. Sequencing was performed using an AB3500 automated sequencer (Applied Biosystems, Illkirch, France).

Statistical analyses were performed using Chi2 tests whenever applicable (expected number >5). Statistical analysis was performed using the SAS program (version 9.4).

## 3. Results

### 3.1. General Statement of Reports 

From 2017 to 2019, 750 online reports were posted on the CNR-LE website: 508 from tertiary care public hospitals and 242 from private laboratories. While the annual numbers of reports from tertiary care public hospitals have been stable since 2017, reports from private laboratories are in constant progression due to the increased number of laboratories joining the network. In 2019, private laboratories represented 47% (130/276) of online reported cases. 

### 3.2. Annual Distribution of Reported Cases

**Figure 1:**
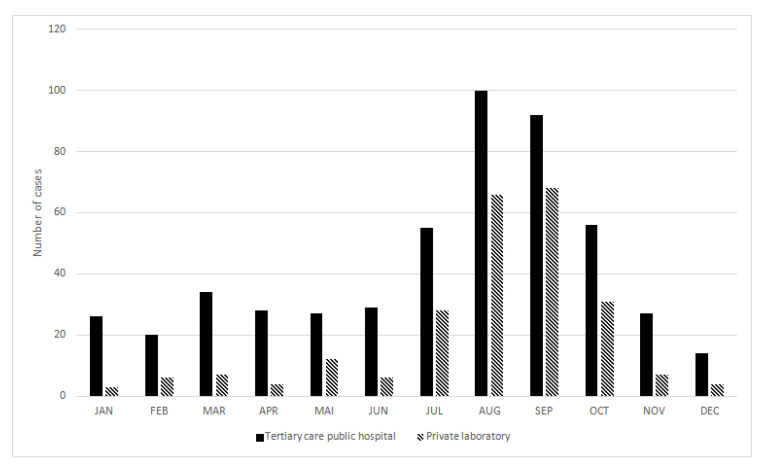
Annual distribution of cryptosporidiosis cases in France from 2017 to 2019.

A similar annual distribution was observed for cases reported by public hospitals and those reported by private laboratories with a large summer peak from July to October, especially in August and September (Figure 1). Cases occurred in summer (July to October) in 71% of immunocompetent patients and in 39% of immunocompromised patients (data not shown). 

### 3.3. Age Distribution of Reported Cases

**Figure 2:**
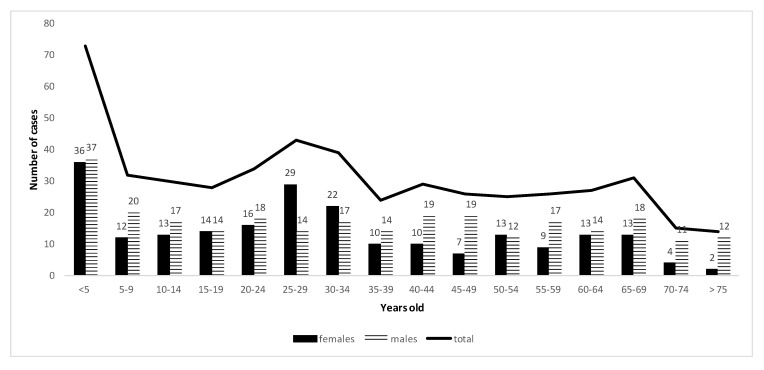
Demography of cryptosporidiosis reported cases from 2017 to 2019 in tertiary care public hospitals in France.

**Figure 3:**
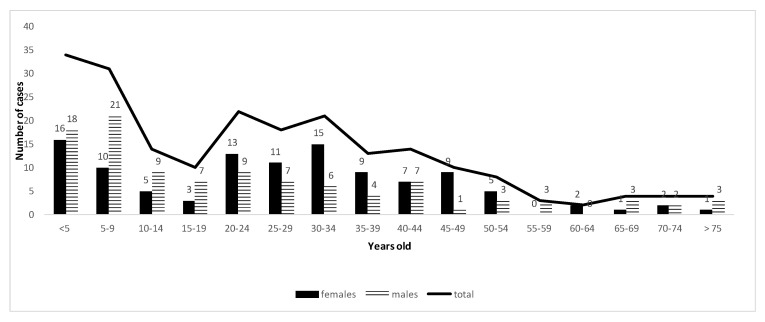
Demography of cryptosporidiosis reported cases from 2017 to 2019 in private laboratories in France.

A similar age distribution of reported cases was observed in public and private laboratories. Two peaks were observed: one concerning children under five years old (especially between 7 and 27 months: data not shown) and another one concerning young adults between 20 and 34 years old. There were similar proportions of female and male cases (45 and 55% respectively); however, in young adults (20–34 years old), female cases were predominant (60 vs. 40%) and in patients older than 55 years, male cases were predominant (72 vs. 28%) (Figure 2 and Figure 3). 

### 3.4. Immune Status of Reported Cases

Of 659 notified cryptosporidiosis cases with informed immune status, 40% (267/659) occurred in immunodeficient patients. Among them, 92% (245/267) were diagnosed in tertiary care public hospitals. The types of immunodeficiency were notified in 258 cases listed in Table 1, predominantly with organ transplantation (53% 136/258). Details on immunosuppressive treatments are presented in Table 2. 

### 3.5. Symptoms and Evolution

Symptoms were recorded in 77% of reported cases (581/750). Details of notified symptoms are presented in Table 3. 

Forty-seven percent of patients (289/620) were hospitalized. Among them, 86% (105/122) of notified hospitalizations were due to cryptosporidiosis. Evolution was recorded in 329 cases and was resolutive in 96% (316/329). Death occurred in 2% (7/329) of patients, all immunocompromised.

### 3.6. Treatment

Of 542 cases with a notified therapy for cryptosporidiosis, 50% were untreated (271/542) and for others, symptomatic treatment was predominant (Table 4). 

### 3.7. Potential Risk Factors

Exposure to risk factors was notified in 40% (300/750) of cases (Table 5). Unbottled water refers to both tap water and wells water. Recreational water refers to bathing water (swimming pool, holiday parks, lake, river, ocean). Additional data focusing on exposure to risk factors in the two dominant age groups is available in Appendix B. 

### 3.8. Cryptosporidium Species and GP60 Subtypes

Among the 750 cases notified between 2017 and 2019, 443 samples were sent to the CNR-LE and 70% (310/443) of isolates were successfully genotyped. *C. parvum* was dominant, representing 72% (222/310), then *C. hominis* in 24% (75/310) of genotyped isolates. Other species were *C. felis* (2%), *C. cuniculus* (>1%), *C. meleagridis* (<1%), *C. canis* (<1%), *C. ubiquitum* (<1%) and *C. erinacei* (<1%). 

**Figure 4:**
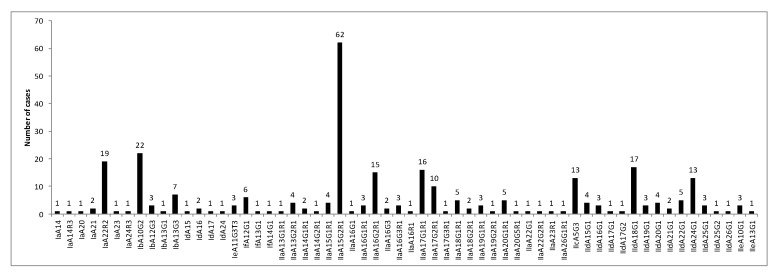
Distribution of *GP60* subtypes of *C. hominis/C. parvum* species among reported cases in France from 2017 to 2019.

*C. parvum* subtype IIaA15G2R1 was represented in 28% (62/222) of *C. parvum* isolates, then IIdA18G1 in 8% (17/222), IIaA17G1R1 in 7% (16/222), IIaA16G2R1 in 7% (15/222), IIdA24G1 in 6% (13/222) and IIcA5G3 in 6% (13/222). Regarding *C. hominis*, the IbA10G2 and IaA22R2 subtypes were dominant representing, respectively, 30% (22/75) and 26% (19/75) of corresponding isolates (Figure 4).

### 3.9. Associations between Clinical Characteristics of Patients and Cryptosporidium Species and GP60 Subtypes 

Associations of parameters between *Cryptosporidium* species and GP60 genotypes are represented in Table 6 and in Appendix A, respectively. No significant relationship was observed between *Cryptosporidium* species and reported items. Women were slightly more infected by *C. hominis* than *C. parvum* (55 vs. 45%) and uncommon species were mainly (61.5%) reported in immunocompromised patients. Regarding risk exposure, direct or indirect contact with potentially contaminated water was recorded in around 50% of cases for both *C. parvum* and *C. hominis*. No recreational water exposure was reported for rare species. Potential human to human transmission was reported for around 20% of cases. Contact with animals was more frequent in *C. hominis* infected patients and rare species. Regarding the results of subtypes represented at least 10 times: the two *C. hominis* dominant subtypes were especially encountered in young children (<5 years old) and in young adults (20–34 years old), especially women; the IaA22R2 subtype strongly infected immunocompetent patients (87.5%) and was mainly reported in the “Grand Est” French region. Diarrhea in close contact was reported in more than 20% of cases for the IIdA18G1, IIdA24G1, IbA10G2, and IaA22R2 subtypes. Direct and indirect water exposure was frequently reported especially for the IbA10G2 subtype. Contact with animals was reported in more than 20% of cases for the IIaA15G2R1 and IIaA16G2R1 subtypes. The IIdA18G1 subtype was mainly encountered in the French region of “Occitanie”. 

## 4. Discussion

Regarding the involvement of the different members of the network, the long-standing participation of tertiary care public hospitals versus private laboratories could be explained by two factors: the relatively small number of hospital centers covering the entire French territory compared to private laboratories, and regular exchanges between these tertiary centers due to frequent meetings during national/international congress or during meetings of the national teaching association of parasitology and mycology. Interestingly, even if few private laboratories have joined the network today, a good coverage of the French territory has been obtained in terms of number of reports; indeed, private laboratories are becoming the main reporters compared to tertiary care centers. Two explanations for the increasing participation of private laboratories could be: (i) a better visibility of the network since its designation as the CNR-LE and (ii) the deployment of molecular genetic diagnostic tools allowing a systematic search for *Cryptosporidium* DNA in stool samples with good sensitivity. The consequences are a constant increase of reported cases from private laboratories since 2017 and, interestingly, cases observed in immunocompetent patients were finally dominant in France, which was not previously observed [24]. In addition, the proportion of cases occurring in immunocompetent patients in France is probably still largely underestimated because of the limited number of private laboratories in the network and because diarrhea etiologies (especially parasitological ones) are rarely investigated in minor forms. This underestimation of *Cryptosporidium* cases in immunocompetent patients is probably encountered worldwide and, finally, immunocompetent cases are often only described through outbreaks, chronic digestive disorders, or in travelers [19,20,25,26]. 

Regarding cases in immunocompromised patients in France, cryptosporidiosis in HIV infected people appeared less predominant than in solid organ transplanted patients (Table 1). Initially, cryptosporidiosis in HIV patients was common and considered as one of the defining agents of the AIDS syndrome [27,28]. The development of anti-retroviral therapy has led to a decrease of cryptosporidiosis in HIV patients especially in developed countries [28] and, concomitantly, the development of solid organ transplantation has led to an increase of cryptosporidiosis in solid organ transplanted patients. As previously described [12], regarding anti-rejection therapy, a large proportion of cryptosporidiosis cases occurred in tacrolimus and/or mycophenolate mofetil regimens (Table 2). It has been reported that the relative risk of developing *Cryptosporidium* infection is lower in cyclosporine-based regimens, compared with tacrolimus-based regimens (odds ratio [OR]: 0.35, 95% confidence interval [CI]: 0.17–0.72, *p* = 0.003) [29]. One explanation could be the enhanced immunosuppressive potential of tacrolimus during cryptosporidiosis since an altered tacrolimus metabolism has been reported in the small intestine consecutive to parasite infection [12,30]. 

Regarding the seasonality of cryptosporidiosis, pronounced seasonal increases of cases have already been described in late spring and late summer–early autumn in Europe [7]. In the United Kingdom (UK), the increased occurrence in spring was mainly due to *C. parvum* and was estimated as a result of environment contamination by oocysts excreted by young animals since the time period coincides with calving and lambing seasons [7,31]. Conversely, the late summer peak was mainly due to *C. hominis* and was attributed to increased travel and exposure to recreational water [7,32]. These high prevalences of *C. parvum* infections in springtime and *C. hominis* infections in summer/autumn were also described in New Zealand, Canada, Ireland, and the Netherlands [28,33,34]. In France, results show that *C. parvum* was mainly responsible for reported cases whatever the season. However, 64% of *C. hominis* reported cases occurred during late summer suggesting that the outcomes already described in the UK (increased travel and exposure to recreational water) could be applicable in France. 

A high prevalence of cryptosporidiosis is documented among children under five years worldwide and among young adults (especially women) in England or in Canada [3,4,7,35]. Potential explanations could be: (i) parents’ contamination from infected children and vice versa; and (ii) poor hygiene, partial protective immunity, ingestion of recreational water and close contact in communities of children [7,18,28]. It seems coherent with the data presented in Appendix B: children were mainly exposed to recreational water, unbottled water, and were in close contact with infected patients (significantly higher than for young adults). Water appeared also predominantly implicated in reported cases of young adults (Appendix B). 

Observed symptoms were classic with, not surprisingly, a strong dominance of digestive disorders (diarrhea, abdominal pain, vomiting, nausea, etc.) [35]. Used treatments were mainly symptomatic and interestingly nitazoxanide was mainly used in immunocompromised patients (89/101), probably due to fear of severe complications, whereas it showed little efficacy on oocyst clearance in immunocompromised patients [7,36]. 

Regarding global proportions of exposure to potential risk factors, water appeared implicated in the majority of notified cases either by direct or indirect consumption, followed by close contact with infected patients, contact with animals and potentially contaminated food ingestion. Similar observations were reported in the literature: in the USA, a waterborne origin was implicated in 41.2% (*n* = 183) of *Cryptosporidium* outbreaks from 2009 to 2017 representing 67.2% (5.015) of cases making it the main mode of transmission. Other more frequent transmission modes were respectively person to person exposure (19.8%) and animal contact (19.4%); foodborne exposure represented only 5% of outbreaks [18]. Similar associated exposures were described in Canada and in Europe [3,7]. 

It has been reported that in industrialized nations such as in European countries, the USA, and Australia, *C. parvum*, and *C. hominis* were equally distributed and *C. hominis* dominated among adults aged between 30 to 40 years old. In Middle Eastern countries, *C. parvum* infections were predominant [7,28,31,37,38]. Zoonotic transmission of *C. parvum* is well documented [31,35,38] and its high prevalence in France was likely associated with the importance of cattle breeding. Interestingly, in the present series of reported cases, contact with animals appeared more frequent in *C. hominis* infected people than in *C. parvum* ones (28% versus 17%). One explanation could be a bias of report because “animal contact” could more easily be understood as pet contact than ruminant contact, leading to potential underestimation of ruminant exposure and consequently an underestimation of global animal contact for *C. parvum* notified cases. To our knowledge, only one publication has reported the detection of *C. hominis* in dog feces [39]. Another explanation could be that animals, and probably pets, could be vectors of cryptosporidiosis through handling. 

Regarding *GP60* subtypes, IIa and IId represented 92% of *C. parvum* genotyped isolates and these subtypes are well known for their zoonotic transmission and are especially encountered in cattle [8,28,40,41]. Among other subtypes, IIcA5G3 subtype was reported (*n* = 13) and interestingly this subtype has only been isolated from humans [42,43,44,45]. The IIdA18G1 subtype appeared mainly represented in the region of “Occitanie” where sheep farming is dominant. It is coherent with the literature since this subtype has already been described in sheep and lambs [46,47]. Not surprisingly, the anthroponotic *C. hominis* IbA10G2 subtype was mainly reported; this subtype is known as the worldwide dominant C. *hominis* subtype [7,20,28,48]. The IbA10G2 subtype was recently reported in cattle and kangaroos in Australia suggesting a potential zoonotic implication, however, as already discussed, handling transmission through pets should also be considered [49,50]. Interestingly, the IaA22R2 subtype was well represented in currently reported cases of *C. hominis* and this subtype is poorly documented in the literature: it was described in one sporadic case in the UK after traveling to Pakistan, and in one child in Nigeria [51,52]. The IaA22R2 subtype appears probably particularly virulent with high observed proportions in immunocompetent (especially young children and young adults) and reported diarrhea in close contact. This subtype was particularly reported in the French “Grand Est” region making us suspect an outbreak, but, unfortunately, no common point was highlighted and water sampling investigation did not reveal the presence of this subtype. 

## 5. Conclusions

From 2017 to 2019, 750 online reports of cryptosporidiosis were made to the CNR-LE. The participation of tertiary care centers is currently optimal in France and, even if the participation of private laboratories is incomplete, it is in constant progression and today accounts for about one half of online reports. The consequences are a better representativeness of the real epidemiology of cryptosporidiosis in France. Results show that, in France, cryptosporidiosis occurred throughout the year but especially in late summer and was predominant in children under 5 years old and in young adults (between 20 and 34 years old). The proportion of declared cases in immunocompetent patients is constantly growing correlated with the increased participation of private laboratories already representing around 60% of notified cases. Among immunocompromised patients, most were solid organ transplanted and among patients on anti-rejection therapy, cryptosporidiosis was mainly described on tacrolimus-based therapy. Treatment was initiated in 50% of cases and was mainly symptomatic; nitazoxanide was frequently used in immunocompromised patients. Of 329 patients with reported clinical evolution, seven patients died (2%) and were exclusively immunocompromised patients. *C. parvum* strongly dominated (72%) the species distribution and especially the widely distributed IIaA15G2R1 zoonotic subtype. The IbA10G2 *C. hominis* subtype was, not surprisingly, dominant but, for the first time to our knowledge, the IaA22R2 subtype appeared also strongly represented and probably highly virulent. All considered, the epidemiology of cryptosporidiosis in France is more and more precise thanks to the participation of an increasing number of collaborating centers within the network. Cryptosporidiosis concerned both immunodeficient and immunocompetent populations but appeared fatal only in immunocompromised patients. These results also suggest a strong implication of environmental contamination in circulating dominant species and subtypes. 

## Figures and Tables

**Table 1:** Distribution of immunodeficiency in cryptosporidiosis cases from 2017 to 2019 in France.

Type of Immunodeficiency	Number of Cases	Proportion
HIV	57	22
Bone marrow transplantation	20	8
Solid organ transplantation	136	53
Malignant pathology	17	7
Primary immune deficiency	7	3
Autoimmune disease	8	3
Malnutrition	2	1
Others *	11	4

* Inflammatory bowel disease, cirrhosis and unspecified.

**Table 2:** Immunosuppressive treatments in solid organ transplanted patients with notified cryptosporidiosis.

Treatment	Number of Cases	Proportion (%)
Cyclosporine	7	5
Cyclosporine + mycophenolate mofetil	15	11
Cyclosporine + tacrolimus	3	2
Tacrolimus	19	14
Tacrolimus + mycophenolate mofetil	73	54
Mycophenolate mofetil	3	2
Others *	16	12

* Everolimus, sirolimus, ibrutinib.

**Table 3:** Recorded symptoms in cryptosporidiosis reported cases.

Symptom	Number of Cases	Proportion (%)
Diarrhea	569	98
Fever	128	22
Nausea	124	21
Weight loss	155	27
Dehydration	86	15
Abdominal pain	257	44
Vomiting	143	25
Respiratory signs	10	2
Increased CRP	57	10
Altered liver function	29	5
Others *	46	8

* Asthenia, myalgia, constipation and bloody stools.

**Table 4:** Cryptosporidiosis treatment in reported cases.

Therapy Used	Number of Cases	Proportion (%)
Oral rehydration	64	24
Parenteral hydration	67	25
Nitazoxanide	101	37
Anti diarrheal	147	54
Antiemetic	19	7
Antibiotic	43	16
Immunosuppresive therapy tapering	33	12
Others *	19	7

* unspecified.

**Table 5:** Potential exposure to risk factors in notified cases.

Exposure	Number	Proportion (%)
Recreational water	144	48
Animal contact	69	23
Close contact with infected patient	75	25
Unbottled water consumption	180	60
Shell consumption	36	12
Raw milk consumption	27	9
Farm cider consumption	6	2

**Table 6:** Associations between *Cryptosporidium* species and symptoms, gender, immune status, and risk factors of notified cases.

	*C. hominis*		*C. parvum*		Other Species		
	(*n* = 75)		(*n* = 222)		(*n* = 13)		*p*-Value
Symptom duration (days)	(*n* = 15)		(*n* = 53)		(*n* = 1)		
Mean (std)	10.0 (5.2)		12.9 (9.4)		15.0 (/)		/
Median (Q1; Q3)	10.0 (6.0; 14.0)		10.0 (7.0; 15.0)		/		
Sex (*n*, %)							
Male	31	44.9	117	54.7	8	61.5	0.2991
Female	38	55.1	97	45.3	5	38.5	
Immune status (*n*, %)							
Immunocompetent	45	66.2	124	63.9	5	38.5	0.155
Immunocompromised	23	33.8	70	36.1	8	61.5	
Diarrhea in close contact (*n*, %)							
Yes	14	28.6	36	26.5	2	20	/
No	35	71.4	100	73.5	8	80	
Water consumption (*n*, %)							
Tap water	19	54.3	56	62.9	2	50	/
Bottled water	16	45.7	33	40.8	2	50	
Shell consumption (*n*, %)							
Yes	4	9.7	10	10.9	2	40	/
No	37	90.3	81	89.1	3	60	
Raw milk consumption (*n*, %)							
Yes	1	2.7	6	7	0	0	/
No	36	97.3	80	93	6	100	
Cider consumption (*n*, %)							
Yes	0	0	3	3.5	0	0	/
No	38	100	84	96.5	5	100	
Contact with animals (*n*, %)							
Yes	12	27.9	18	16.8	1	25	/
No	31	72.1	89	83.2	3	75	
Recreational water exposure (*n*, %)							
Yes	19	48.7	50	50	0	0	/
No	20	51.3	50	50	5	100	

## Appendix A

**Table A1:** Notified parameters of epidemiology and potential risk factors in the main subtypes.

Subtype	IIaA15G2R1 (*n* = 62)		IIdA18G1 (*n* = 17)		IIaA17G1R1 (*n* = 16)		IIaA16G2R1 (*n* = 15)		IIdA24G1 (*n* = 13)		IIcA5G3 (*n* = 13)		IbA10G2 (*n* = 22)		IaA22R2 (*n* = 19)	
**Symptom duration (days)**	**(*n* = 12)**		**(*n* = 6)**		**(*n* = 3)**		**(*n* = 4)**		**(*n* = 2)**		**(*n* = 4)**		**(*n* = 2)**		**(*n* = 6)**	
Mean (std)	14.6 (8.8)		10.8 (6.0)		9.0 (2.7)		21.5 (25.8)		12.5 (7.8)		9.8 (6.0)		10.5 (5.0)		8.0 (3.3)	
Median (min; max)	10.0 (5.0; 30.0)		11.0 (3.0; 18.0)		8.0 (7.0; 12.0)		10.5 (5.0; 60.0)		12.5 (7.0; 18.0)		8.5 (4.0; 8.0)		10.5 (7.0; 14.0)		8.5 (3.0; 12.0)	
**Age**																
<5 years	7	12.1	2	11.7	2	13.3	1	6.7	3	23.1	2	14.4	4	18.2	5	26.3
5–9 years	7	12.1	1	5.8	3	18.7	3	20.0	0	0	2	15.4	2	9.1	1	5.3
10–14 years	2	3.5	1	5.8	1	6.3	1	6.7	0	0	0	0	2	9.1	1	5.3
15–19 years	2	3.5	0	0	1	6.3	1	6.7	2	15.4	1	7.7	0	0	1	5.3
20–24 years	7	12.1	2	11.8	1	6.3	2	13.3	1	7.7	1	7.7	1	4.6	4	21.0
25–29 years	5	8.6	2	11.8	1	6.3	1	6.7	1	7.7	0	0	3	13.6	0	0
30–34 years	6	10.3	2	11.8	1	6.3	2	13.3	0	0	0	0	1	4.6	3	15.8
35–39 years	3	5.2	1	5.8	0	0	1	6.7	0	0	1	7.7	0	0	0	0
40–44 years	0	0	0	0	0	0	2	13.3	2	15.4	0	0	2	9.1	1	5.3
45–49 years	4	6.9	0	0	0	0	1	6.7	0	0	0	0	1	4.6	0	0
50–54 years	2	3.5	2	11.8	3	18.7	0	0	0	0	2	15.4	0	0	0	0
55–59 years	2	3.5	2	11.8	1	6.3	0	0	0	0	2	15.4	0	0	0	0
60–64 years	1	1.7	1	5.8	1	6.3	0	0	0	0	0	0	1	4.6	1	5.3
65–69 years	6	10.3	1	5.8	0	0	0	0	2	15.4	0	0	1	4.6	0	0
70–74 years	3	5.2	0	0	0	0	0	0	1	7.7	0	0	2	9.1	0	0
>75 years	1	1.7	0	0	0	0	0	0	1	7.7	0	0	2	9.1	0	0
**Sex (*n*, %)**																
Male	35	59.3	8	50.0	8	53.3	9	60	6	46.2	2	40	8	40	7	36.8
Female	24	40.7	8	50.0	7	46.7	6	40	7	53.8	5	60	12	60	12	63.2
**Immune status (*n*, %)**																
Immunocompetent	34	61.8	7	46.7	7	63.6	6	54.5	8	66.7	7	70	11	57.9	14	87.5
Immunocompromised	21	38.2	8	53.4	4	36.4	5	45.5	4	33.3	3	30	8	42.1	2	12.5
**Sympomatic (*n*, %)**																
Yes	53	98.2	16	100	11	100	13	100	12	92.3	10	100	16	94.1	16	100
No	1	1.8	0	0	0	0	0	0	1	7.7	0	0	1	5.9	0	0
**Diarrhea in close contact (*n*, %)**																
Yes	7	17	3	25	0	0	1	16.7	3	37.5	1	16.7	2	22.2	5	35.7
No	34	83	9	75	6	100	5	83.3	5	62.5	5	83.3	7	77.8	9	64.3
**Water consumption (*n*, %)**																
Tap water	16	57.1	4	66.7	1	50	3	50	5	83.3	1	33.3	7	77.8	6	66.7
Bottled water	12	42.9	2	33.3	1	50	3	50	1	16.7	2	77.7	2	22.2	3	33.3
**Shell consumption (*n*, %)**																
Yes	1	3.8	2	28.6	0	0	0	0	1	20	0	0	1	10	1	8.3
No	25	96.2	5	71.4	3	100	5	100	4	80	3	100	9	90	11	91.7
**Raw milk consumption (*n*, %)**																
Yes	0	0	0	0	0	0	0	0	1	20	0	0	0	0	0	0
No	24	100	6	100	3	100	5	100	4	80	3	100	8	100	11	100
**Cider consumption (*n*, %)**																
Yes	0	0	0	0	0	0	0	0	1	20	0	0	0	0	0	0
No	26	100	5	100	3	100	7	100	4	80	3	100	9	100	10	100
**Contact with animals (*n*, %)**																
Yes	8	22.2	0	0	0	0	2	40	0	0	0	0	2	20	2	15.4
No	28	77.8	8	100	3	100	3	60	5	100	4	100	8	80	11	84.6
**Recreational water exposure (*n*, %)**																
Yes	23	63.9	1	14.3	0	0	4	66.7	2	40	1	16.7	6	75	4	40
No	13	36.1	6	85.7	2	100	2	33.3	3	60	5	83.3	2	25	6	60
**French Regions (*n*, %)**																
Auvergne-Rhône-Alpes	3	5.9	1	7.7	1	8.3	1	9.1	0	0	0	0	0	0	0	0
Bourgogne-Franche-Comté	2	3.9	0	0	1	8.3	0	0	0	0	1	12.5	1	7.1	0	0
Bretagne	8	15.7	1	7.7	0	0	1	9.1	0	0	0	0	2	14.3	0	0
Centre-Val de Loire	1	2.0	0	0	0	0	0	0	0	0	0	0	0	0	0	0
Grand Est	17	33.3	2	15.4	1	8.3	3	27.3	0	0	2	25	3	21.4	8	57.1
Hauts-de-France	2	3.9	1	7.7	1	8.3	1	9.1	1	20	2	25	0	0	0	0
Ile-de-France	4	7.8	0	0	3	25	1	9.1	0	0	0	0	1	7.1	0	0
Normandie	6	11.8	3	23.1	1	8.3	2	18.2	3	60	1	12.5	2	14.3	2	14.3
Nouvelle-Aquitaine	3	5.9	0	0	0	0	0	0	0	0	0	0	2	14.3	3	21.5
Occitanie	3	5.9	4	30.8	2	16.7	1	9.1	0	0	0	0	0	0	1	7.1
Pays de la Loire	2	3.9	0	0	1	8.3	0	0	0	0	1	12.5	1	7.1	0	0
Provence-Alpes-Côte-D’azur	0	0	1	7.7	1	8.3	1	9.1	1	20	1	12.5	2	14.3	0	0

## Appendix B

**Table A2:** Associations between risk factors and most represented age populations.

Exposure (*n*, %)	<5 Years		20–34 Years Old		*p*-Value
	*n*	%	*n*	%	
Recreational water	34	67	36	48	0.27
Animal contact	7	15	20	21	0.45
Close contact with infected patient	29	43	24	20	0.01 *
Unbottled water consumption	24	53	51	70	0.75
Shell consumption	2	4	16	21	/
Raw milk consumption	1	1	10	13	/
Farm cider consumption	0	0	0	0	/

* Significant *p*-value (<0.05).

## Appendix C

### French National Network on Surveillance of Human Cryptosporidiosis

Anne Debourgogne, Adela Angoulvant, Patrice Agnamey, Julie bonhomme, Brigitte Degeilh, Cathy Chemla, Cécile Garnaud, Coralie Lollivier, Cécile Angebault, Pascal Delaunay, Guillaume Desoubeaux, Emilie Fréalle, Frédéric Grenouillet, Florent Morio, Françoise Botterel, Ghania Belkadi, Hélène Yera, Gilles Nevez, Xavier Iriart, Isabelle Accoceberry, Julie Brunet, Marc Thellier, Meja Rabodonirina, Milene Sasso, Charline Miossec, Murielle Nicolas, Nicole Desbois, Philippe Poirier, Christelle Pomares, Sandrine Houze, Céline Nourrisson, Gabriella Certad, Gladys Robert, Florence Robert-Gangneux, Yohann Le govic, Anne Pauline Bellanger, Franck Labbe, Frédéric Janvier, Céline Damiani, Marie Hélène Rodier, Christine Schuttler, Marie Laure Darde, Luc De Gentile, Marie Pierre Hayette, Olivier Duquesnoy, Pierre Flori, Yvon Sterkers, Jean Philippe Duvert, Jean Philippe Lemoine, Pierre Marty, Bernard Levy, Edith Mazars, Patrick Bastien, Thomas Gueudet, Christine Rieder, Marie-odette Guy, Stéphane Larreche, Sophie Lesthelle, Isabelle Villena, Dominique Aubert, Stéphane Lastere, Estelle Cateau, Claudie Leclair, Alida Minoza, Kévin Brunet, Marie-Elisabeth Bougnoux, Nathalie Kapel, Eric Dannaoui, Noémie Coron, Alexis Valentin, Laura courtellemont, Joséphine Dorin, Anne Totet, Laura Verdurme, Nawel Ait Ammar, Alicia Moreno Sabater, Jean Menotti, Pascal Millet, Alexander Pfaf, Denis Blanchet, Meja Rabodonirina, Magalie Demar, Emmanuel Dutoit, Renaud Piarroux, Emilie Sitterle, Denis Magne, Samsa Hamane, Antoine Berry, Cecile Ramade
